# Magnetoencephalography with optically pumped magnetometers (OPM-MEG): the next generation of functional neuroimaging

**DOI:** 10.1016/j.tins.2022.05.008

**Published:** 2022-06-30

**Authors:** Matthew J. Brookes, James Leggett, Molly Rea, Ryan M. Hill, Niall Holmes, Elena Boto, Richard Bowtell

**Affiliations:** 1 Sir Peter Mansfield Imaging Centre, School of Physics and Astronomy, University of Nottingham, University Park, Nottingham, NG7 2RD, UK

## Abstract

Magnetoencephalography (MEG) measures human brain function via assessment of the magnetic fields generated by electrical activity in neurons. Despite providing high-quality spatiotemporal maps of electrophysiological activity, current MEG instrumentation is limited by cumbersome field sensing technologies, resulting in major barriers to utility. Here, we review a new generation of MEG technology that is beginning to lift many of these barriers. By exploiting quantum sensors, known as optically pumped magnetometers (OPMs), ‘OPM-MEG’ has the potential to dramatically outperform the current state of the art, promising enhanced data quality (better sensitivity and spatial resolution), adaptability to any head size/shape (from babies to adults), motion robustness (participants can move freely during scanning), and a less complex imaging platform (without reliance on cryogenics). We discuss the current state of this emerging technique and describe its far-reaching implications for neuroscience.

## Brain imaging via MEG

MEG [1] is a noninvasive method allowing real-time imaging of brain function. The technique is based on measurement of magnetic fields outside the head, which are generated (primarily) by synchronous dendritic current flow through neuronal assemblies. Mathematical modelling of these fields enables the generation of 3D images (termed source localisation) showing how electrical activity changes, moment-to-moment, as the brain responds to various experimental scenarios or cognitive demands. MEG has temporal resolution in the millisecond range and spatial resolution of ~2–5 mm [2]. With these characteristics, MEG has many advantages over other functional imaging modalities [3], including functional magnetic resonance imaging (fMRI), which is limited to haemodynamic metrics and has limited temporal resolution, and electroencephalography (EEG), the spatial resolution of which is limited by distortions in electrical potential caused by the skull. Consequently, MEG has become an important part of the neuroscientific toolbox for noninvasive imaging. However, the present generation of MEG scanners has significant limitations, which hamper their utility.

The fundamental problem limiting MEG’s applicability is that, to gain sufficient sensitivity to measure the small (~100 fT) magnetic fields generated by the brain, current MEG systems (which are housed in a magnetically shielded environment to suppress background fields) employ pick-up coils that are coupled to superconducting quantum interference devices (SQUIDs) [4,5]. These sensors typically require cooling to ~4 K (−269°C). This, in turn, means sensors are bathed in liquid helium and a vacuum is maintained between the sensors and the participant’s scalp, for thermal insulation. Sensors must consequently be formed into a fixed array around the scalp.

These design considerations underlie many of the limitations of MEG. First, the fixed array means that participants must remain still relative to the sensors throughout data acquisition. Coping with the MEG environment is consequently challenging for some participants. Second, the MEG signal strength decreases with the square of distance from the source (the inverse square law); the requirement for thermal insulation between the scalp and the sensor limits proximity (the closest a sensor can be to the scalp is ~2 cm) and this limits signal strength. The need for rigidity also means that a ‘one size fits all’ MEG helmet, built to fit ~95% of adults, is used. In practice, this means that the helmet is designed for someone with a relatively large head; most people will not fit the helmet perfectly and the gap between the scalp and the helmet will vary across the head. This results in inhomogeneous coverage. For those with smaller heads this effect is amplified and it is hard to simultaneously obtain uniform coverage and high sensitivity in infants. Finally, the complexities of the system make scanners costly to buy and maintain and the need for cryogenics means either a constant supply of liquid helium or a local helium reliquefier is needed.

In recent years, the MEG field has seen the introduction of a new magnetic field-sensing technology. OPMs [6] are magnetic-field sensors that offer sensitivity comparable to SQUIDs without relying on cryogenic cooling. This has resulted in the evolution of new MEG systems (e.g., [7]) and, though still nascent technology, ‘OPM-MEG’ scanners are beginning to out-perform the current state of the art, offering higher quality data, improved uniformity of coverage, motion robustness, and lower system complexity. In this review, we outline the current state of OPMMEG, describing the technology, its advantages, and limitations. We also review current literature and speculate on where this new technology could take the neuroimaging field.

## The technical advantages of OPMs

**Figure 1.**
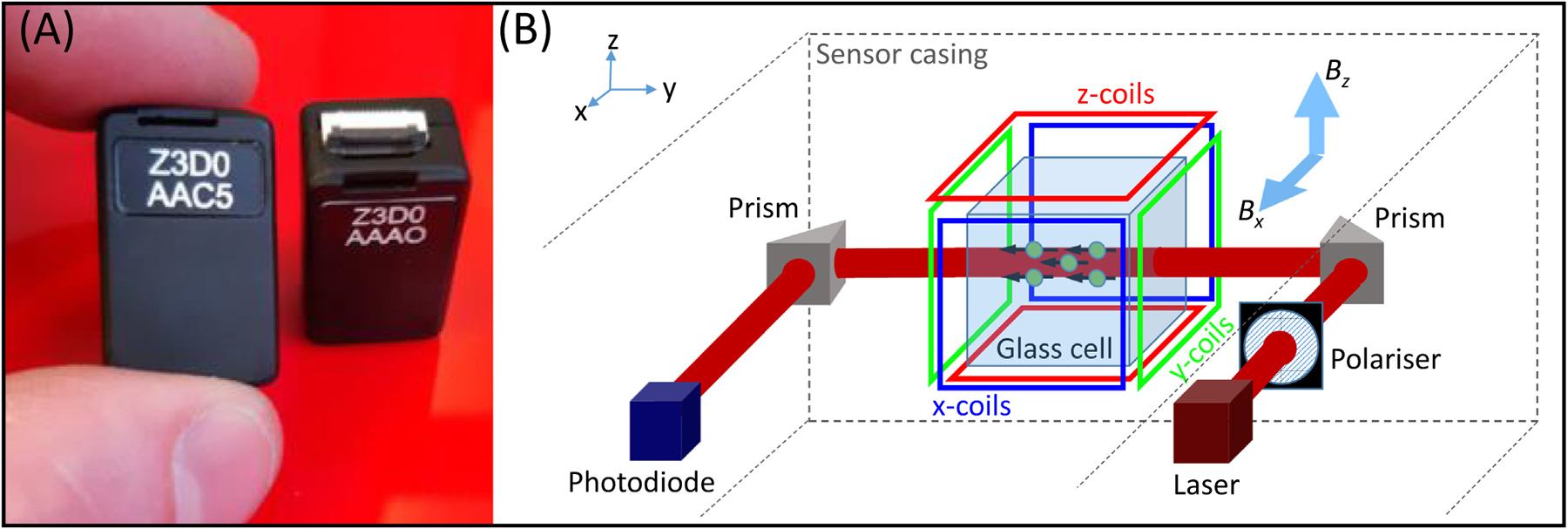
Optically pumped magnetometers (OPMs). (A) Two OPM sensors, made by QuSpin Inc. (www.quspin.com). Sensors are approximately the size and shape of a (2 × 4) Lego brick. (B) A schematic diagram of the inside of an OPM, showing the component parts. Laser light is directed through a glass cell to interact with atoms in a ^87^Rb vapour. Coils placed around the cell enable control of the magnetic field within the cell, along all three Cartesian axes. With the laser beam oriented in the y direction, fields oriented in both x and z (*B_x_* and *B_z_* respectively) can be measured.

OPMs use the quantum properties of atoms to sense local magnetic fields [8]. OPMs have been in development for several decades, with recent years seeing marked improvements in sensitivity and miniaturisation. The devices that have become popular for MEG are small, self-contained units, approximately the size and shape of a (2 × 4) Lego brick (Figure 1A) [9–11]. Each unit comprises a glass cell containing an atomic vapour (alkali atoms, usually ^87^Rb), a laser and associated optics to project polarised laser light through the cell, a set of electromagnetic coils for field control within the cell, and a photodiode for detection of light passing through the vapour (Figure 1B).

With the atoms ‘pumped’ by the laser into a specific quantum state, the atomic vapour becomes magnetised and interacts with any external magnetic field (e.g., the neuromagnetic field) that passes through the sensor. Such interactions modulate the amount of light passing through the vapour and the field magnitude can be inferred by measurement at the photodiode (Box 1). Magnetic fields can be measured in two perpendicular orientations (in the plane perpendicular to the laser beam; Figure 1B) with a noise floor of around 7–10 fT/sqrt (Hz). [For comparison, most SQUIDs have a noise floor of around 2–5 fT/sqrt (Hz).]

**Figure 2.**
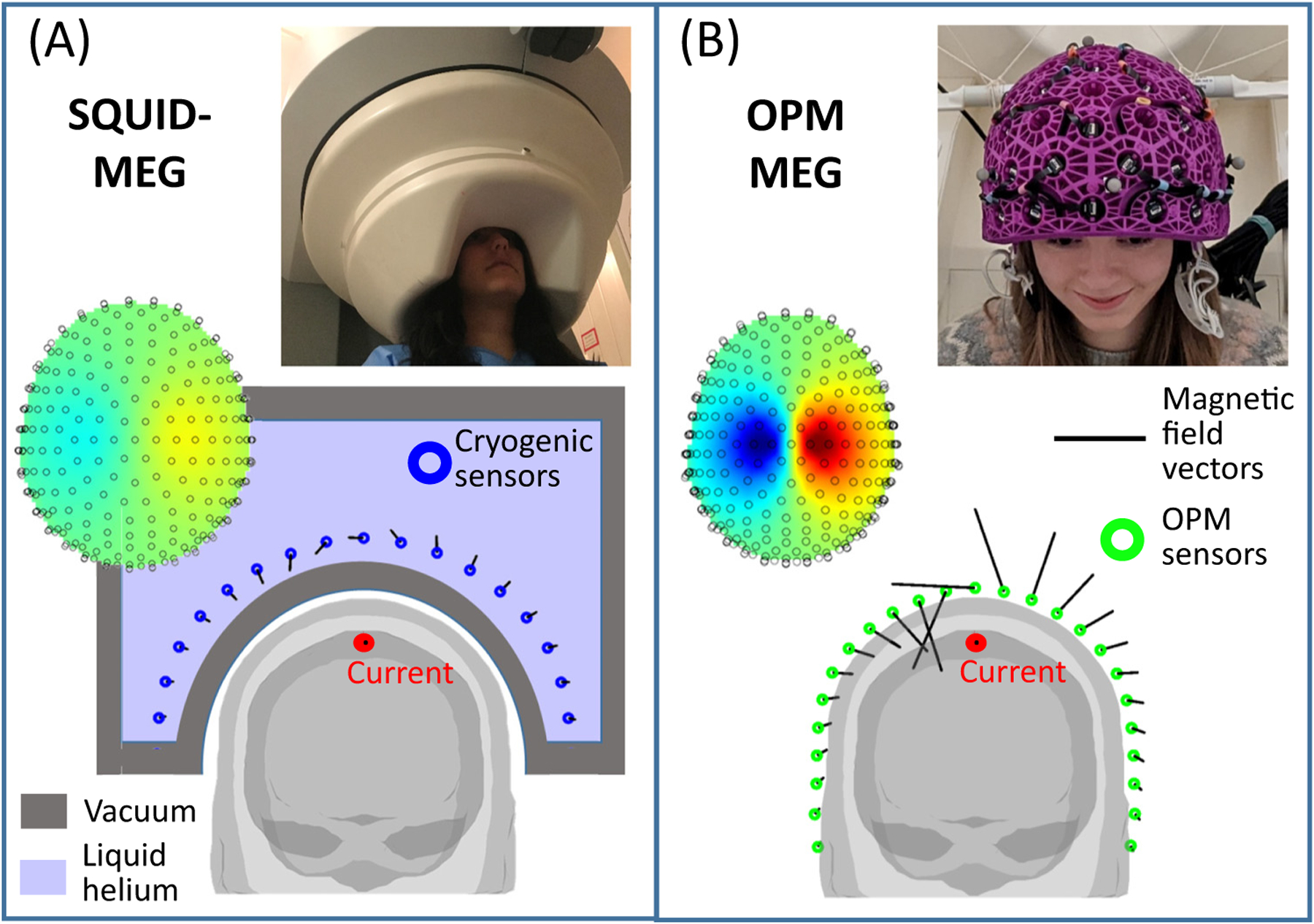
Advantages of optically pumped magnetometer (OPM)-magnetoencephalography (MEG) compared with conventional MEG. (A) A schematic representation of conventional MEG [superconducting quantum interference device (SQUID)-MEG]. A participant sits with their head in a static helmet (see inset photo, adapted from [16]), containing an array of field sensors (blue circles). Sensors require cryogenic cooling and are consequently bathed in liquid helium. The requirement for thermal insulation (provided by a vacuum, shown in grey) limits sensor proximity to the head, hence the size of the measured magnetic field (represented by the length of the black lines) is limited. For participants with smaller heads (and particularly infants or babies) the sensors would be even further away and, consequently, the signal-to-noise ratio even lower. (B) A schematic representation of OPM-MEG. OPMs do not require cryogenic cooling and so can be mounted flexibly in a lightweight helmet (see inset photo, adapted from [54]) that can be made to fit any head shape. Because sensors are closer to the head compared with conventional MEG, the measured fields (black lines) are larger, increasing sensitivity. In addition, closer proximity allows denser sampling of focal field patterns (examples of which are also shown in the insets), which increases spatial resolution.

OPMs of this type usually operate in the spin-exchange relaxation-free (SERF) regime [6], which requires the ^87^Rb-vapour to be heated to ~150°C. However, thermal insulation (e.g., aerogel) allows the cell to be placed just a few millimetres from the scalp, compared with ~2 cm or more in cryogenic MEG. The reduced sensor-scalp separation has two effects (Figure 2). First, the magnitude of measured magnetic field vectors is larger when sensors are placed on the scalp surface, compared with when sensors are placed at the distance required for cryogenic MEG. Simulations [12,13] show that this effect provides a four- to fivefold signal enhancement in many cortical areas. As a result of the nonlinearity in the inverse square law, this advantage declines with depth (e.g., to a factor of ~2 for deeper cortical regions [12] and likely lower in subcortical structures). Nevertheless, there is potential for enhanced signal strength across the whole brain. If the noise floors of OPMs and SQUIDs were equal, this would result in a similar increase in signal-to-noise ratio (SNR) for OPM-based systems. In practice, the noise floor of OPMs currently remains higher than that of a SQUID, nevertheless increased SNR for cortical sources has been realised experimentally; for example, in a healthy adult, the SNR of evoked responses in the sensory cortex generated by median nerve stimulation was improved by a factor of ~2 using OPMs compared with SQUID measurements [14]. In deeper sources, the advantage of proximity is (at present) negated by the higher noise of an OPM. That said, OPMs have been used to image subcortical structures, notably the hippocampus [15]. Second, the enhanced proximity of the sensors means that measured field patterns are more ‘focal’ (Figure 2). This allows better separation of field patterns arising from spatially separate current sources in the brain. Simulations [13] show that the correlations between field patterns generated by separate sources are reduced approximately threefold by moving sensors closer to the scalp. This should translate to improved spatial resolution [16]. In support of this, recent simulation work [17] showed that a densely packed OPM array over a patch of cortex can localise electrophysiological brain responses at unprecedented resolution for a noninvasive device (a process the authors term ‘magnetocorticography’). However, careful system calibration was a significant factor. Again, it is important to note that this is a function of depth, with the largest gains in superficial cortical areas (i.e., close to the skull). In sum, OPM-MEG offers two fundamental advantages in performance over current MEG: higher sensitivity and enhanced spatial resolution. Both effects are notable in all subjects, but are particularly pronounced when imaging individuals with smaller heads (e.g., infants).

Apart from permitting greater proximity to the scalp, OPMs also allow the design of bespoke sensor arrays; this means that the array can be tailored to best match the participant, or the experiment that is to be carried out. For example, helmets housing the sensors can come in different sizes [18], or can be made bespoke for a single participant [14], thus ensuring uniform coverage and adaptability to scan almost anyone, from babies to adults. Arrays can be designed to target specific brain regions with high sensor density, for example, if high spatial resolution is desired in a specific area (recently published examples include the language network [19], hippocampus [20], and cerebellum [21]). It is also becoming apparent that OPM use is not limited to the brain, with arrays also having been used to measure electrophysiological signals in the muscles [22], peripheral nerves [23], spinal cord [24], retina [25], and the foetus [26]. Another advantage is that, whereas SQUIDs typically measure the magnetic field in one orientation (usually radial to the scalp), OPMs can simultaneously measure components of the magnetic field vector along multiple directions [27]. The tangential components of the neuromagnetic field are smaller than the radial components [13,28] but still contain useful information. Studies have shown that the addition of tangential components offers advantages when trying to differentiate fields from within the brain from those originating outside the head (i.e., interference) [29] and when the number of sensors is limited [28]. Thus, the flexibility of OPMs to make bespoke arrays, and to offer multidimensional magnetic field metrics, is bringing about a change in MEG capabilities.

## Towards wearability

OPMs reached sensitivities comparable with SQUIDs in the early years of the 21st century, with the first MEG measurements (of the auditory evoked response) published in 2006 [30]. However, the experimental set-up was laboratory-based and the large footprint of the OPMs negated the possibility of simultaneous measurements from a large number of sites on the scalp. Nevertheless, the potential was recognised and there followed a period of miniaturisation, with lightweight OPMs emerging around the early 2010s. By 2010, a small SERF magnetometer had been used to measure evoked responses from median nerve and auditory stimulation [10]. By 2013, the same group had demonstrated a multisensor OPM array [7]. Other demonstrations included the use of a chip-scale OPM to measure evoked and oscillatory neuromagnetic effects [11] and measurement of the modulation of occipital alpha oscillations by opening and closing the eyes [31]. A significant step forward came with the introduction of commercial OPMs, by QuSpin Inc. in 2016, allowing the neuroimaging community to begin to build OPM-MEG systems. This drove further progress and soon multiple groups had begun to demonstrate the promise of robust and reliable microfabricated OPMs for MEG measurement (e.g., [14,32–34]).

A major limitation of conventional MEG (and fMRI) is limited tolerance to participant movement. Because the sensor array is fixed, any motion of the participant relative to the array causes changes in signal amplitude and SNR (as brain regions get closer to, or further from, the sensors) and spatial blurring of the field topography. Consequently, MEG signals from conventional scanners become distorted, in time and space. Recent years have seen the development of algorithms to measure (in real time) and correct (in post-processing) such artefacts (e.g., [35–38]) and the importance of these methods, particularly in paediatric imaging, is well recognised [39–41]; a simulation study [42] has shown that, whilst head movement results in a significant degradation in spatial accuracy, with appropriate compensation, accuracy can be restored to premovement levels even in the presence of small (e.g., 2–3 cm) movements. These techniques can allow high-fidelity MEG acquisition in infants and patient groups who find it hard to remain still. However, other studies have suggested complications due to changes in SNR as sources move relative to the sensors, potentially placing upper limits on the magnitude of movement that can be compensated [43]. Most importantly, even with movement compensation, successful MEG measurement relies on the subject’s head remaining inside the helmet and this places a hard limit on the allowable head movement. For adults, movement is physically restricted (i.e., a movement of more than a few centimetres would cause the subject to hit their head on the helmet), which limits the ability to perform naturalistic tasks. In infants, the requirement to remain within the helmet can be met with training [44], but many infants still find the unnatural environment difficult to tolerate and again this restricts experimental paradigms. This has been one of the major limitations of MEG/fMRI compared with EEG or functional near infra-red spectroscopy (fNIRS) [45], both of which involve wearable instrumentation where movement is not curtailed by a static helmet (or for fMRI, an enclosed scanner).

By contrast, the lightweight nature of OPMs means that sensors, mounted in a suitable helmet (Box 2), can move with the head. Consequently, the MEG scanner can become a wearable device, allowing (in principle) any degree of motion throughout a scan. In practice however, it is not that simple. OPMs are ‘vector’ magnetometers and are directionally sensitive; meaning if they move relative to any remnant (temporally static) magnetic field, they will measure a field change. This leads to artefacts in the data (i.e., fluctuations in the measured signal not generated by the brain). More importantly, those field changes can also render OPMs inoperable because the dynamic range (i.e., the range of field an OPM can tolerate) is small. Indeed, in a ‘typical’ MEG shielded environment (e.g., with background field of ~30 nT), even a head rotation of ~4° could render the OPMs inoperable. In addition, temporally changing fields, for example, caused by nearby equipment or infrastructure, can also cause interference and send OPMs outside their dynamic range (even if the OPMs remain stationary). For these reasons, the success of OPM-MEG, and in particular the ambition for a wearable system, is highly dependent on ‘magnetic shielding’ (i.e., the ability to remove background magnetic fields).

Recent years have seen rapid progress in the design of ‘OPM-optimised’ magnetic shielding (Box 3). Such shields reduce the magnetic field surrounding the array in two ways: first, the OPM system is surrounded by layers of high permeability (mu-metal, a nickel-iron alloy) and high conductivity (aluminium or copper) material, collectively termed passive shielding. Second, reference sensors within the room measure the remnant fields and electromagnetic coils generate equal and opposite fields to those measured, thus cancelling them out [35,46,47], termed active shielding. The result of combining these two shielding methods is that the participant’s head sits in a (close-to) zero-field environment and, consequently, movement does not affect the field seen by the OPMs. Of course, shielding can never be perfect, but data show that, using current techniques, the static magnetic field can be reduced from ~60 μT with no shielding (the Earth’s field) to ~5 nT with ‘passive’ shielding, and to ~200 pT with both passive and active shielding (a shielding factor of ~300 000). In addition, low frequency (<3 Hz) temporally varying interference is also minimised. This means high quality MEG data can be acquired and, even if participants move, OPMs continue to function [16].

The ability to move opens up possibilities to record OPM-MEG data in experimental settings that allow participants to interact with their environment in ways that are not feasible using conventional MEG or fMRI. This wearability has now been shown a number of times: in a first demonstration [16], participants were scanned whilst drinking a cup of tea and playing a ball game (bouncing a table tennis ball on a bat). The retinotopic organisation of the visual cortex was also demonstrated [46], but rather than a visual stimulus being moved around a participant’s field of view, the participant moved their head to view a static stimulus from multiple angles. The ability to move has enabled new types of stimulation to be used, for example, using a virtual reality headset to immerse a participant in a virtual world [48]; this opens up possibilities for new paradigms (e.g., spatial navigation). Participants have been scanned successfully whilst playing interactive computer games that require natural movement [18] and whilst learning to play a musical instrument [18], demonstrating the potential of OPM-MEG for motor learning experiments. Most recently, the auditory cortex was localised in participants who were standing and/or making large movements (in this case, in the absence of active shielding) [49]. This also paves the way for new opportunities, for example, studies in individuals with movement disorders.

It is important to note that measurements of brain function during naturalistic movements are not unique to OPM-MEG; indeed they are possible using EEG and fNIRS. However, compared with EEG, even conventional MEG offers improved spatial precision [3] and OPM-MEG offers additional improvements in both spatial resolution and sensitivity [50]. Further, MEG has approximately tenfold lower sensitivity to artefacts from muscles during movement compared with EEG [50]. This latter point is important, since in a wearable system where movement is encouraged, muscle artefact can often obfuscate brain signals at frequencies above ~20 Hz. Compared with fNIRS (which measures a haemodynamic response) OPM-MEG has both improved spatial and temporal resolution. For these reasons, OPM-MEG has the potential to become the method of choice for investigation of brain function in humans during naturalistic interactive experimental paradigms.

## Neuroscientific applications

OPM-MEG has now been used to measure many of the electrophysiological phenomena commonly reported using MEG and EEG. For example, assessment of evoked responses to sensory stimuli of various modalities is commonplace, with OPMs providing high fidelity metrics (see [51] for an excellent example). Similarly, neural oscillations have been measured across multiple frequency bands, again with high SNR (see [34] for an example). Epileptiform activity has been characterised using wearable OPM-MEG devices [52,53], demonstrating the promise for future application in the clinic. Studies using whole-head systems have shown that wearable MEG performance can surpass that of conventional systems, even when using a lower channel count. For example, recent work [54] contrasted a 50-channel wearable OPM-MEG system to a 275-channel cryogenic MEG system, with favourable results. The introduction of wearable, whole-head systems has enabled OPM-MEG to measure functional connectivity [55], with electrophysiological networks clearly delineated. The prospects of using wearable MEG for brain computer interfacing (BCI) have also been demonstrated [56]: in a ‘mind-spelling’ task, participants were asked to look at a keyboard presented on a screen and fix their gaze on the letter they wished to type. OPM-MEG signals were then processed using a machine learning algorithm, to determine which letter the participant was gazing at. Letters were correctly identified in 97.7% of trials. This not only demonstrates the potential for future application in BCI, but also showcases the high-fidelity data that can be acquired using OPM-MEG devices.

One limiting factor of OPMs is their performance at low frequency; there are three reasons for this. First, the inherent OPM sensor noise increases for low frequencies. Second, OPMs are magnetometers, which are sensitive to distant interference sources, and the lower the frequency of interference, the harder it is to shield. Third, for wearable systems, movement, even in very low background fields, will induce a degree of artefact and such artefacts usually manifest at low frequencies. Nevertheless, in recent work, a wearable OPM system was successfully used for cortical tracking of speech [57] and results showed that the MEG signals tracked the rhythmicity of phrases (0.2–1.5 Hz signals) and words (2–8 Hz signals) with reconstruction accuracy close to that previously reported in conventional MEG studies, suggesting that OPM-MEG is well suited to measure brain activity at frequencies below 4 Hz. Relatedly, theta (4–8 Hz) oscillations in the hippocampus were successfully measured [20] using a unique design of OPM-array where, to improve array sensitivity, an OPM was placed in the participant’s mouth, demonstrating again the flexibility of array design, as well as sensitivity to low frequencies.

One area where OPMs have a distinct advantage is in paediatric measurements, where the adaptability of helmet design, coupled with motion tolerance, means that OPMs can be made to work in cohorts of infants/children where conventional MEG (and indeed other imaging modalities) are difficult to deploy. This field is in its infancy, with relatively few published demonstrations. Nevertheless, the potential is evidenced. Initial work [18] used 12 OPMs, mounted in a modified bike helmet, to measure the electrophysiological response to parental touch (i.e., a parent gently stroking the hand of their child). Successful measurement of beta band power reduction due to sensory stimulation was measured and localised to primary somatosensory cortex, in both a 2-year-old and a 5-year-old. Supporting this, a more recent study [58] used the first operational array of triaxial OPMs (i.e., sensors that measure magnetic field along three orthogonal axes, simultaneously) to measure the same beta band effects in a 5-year-old. This study also showed that triaxial measurements offer significant advantages in terms of cortical coverage in infants and children, compared with the more widely used dual-axis OPMs. Perhaps most significantly, a recent study [53] aimed to measure interictal epileptic discharges in children, aged 5–9 years, using a wearable OPM array, comparing the results with measures from the same individuals in a SQUID system. Results showed that the SNR obtainable using OPMs was significantly higher than that using SQUID-MEG. Thus, although these are early days for application of OPM-MEG in infants/children, it is becoming clear that paediatric functional imaging is one area in which OPM-MEG shows promise.

## Challenges

OPM-MEG is relatively new and the remaining challenges should be fully appreciated. From a technical point of view, the current generation of OPMs have not yet reached the noise floor of the SQUID. Whilst for most cortical sources the higher noise of an OPM is (more than) compensated by sensor proximity (giving OPMs an SNR advantage), for deep sources SQUIDs may still offer greater SNR (since the proximity compensation is diminished). This said, miniaturised OPMs are a recent development and their performance has improved significantly over recent years; there is no fundamental reason that OPM-MEG cannot surpass the sensitivity of conventional MEG for deep, as well as cortical structures. Also relating to OPM-design, a significant challenge is the heat generated by the sensors. For current systems, a combination of judicious helmet design and insulation (aerogel) is keeping the temperature on the scalp comfortable. However, as systems move towards higher numbers of sensors, a greater amount of heat will be dissipated, with the potential to become uncomfortable for the participant. Future OPM-MEG systems may then require active cooling (e.g., cold air or water forced through the helmet [59]). In this context, a new generation of OPMs based on paramagnetic resonance of helium-4, which do not require either heating or cooling, shows promise [60–62]. On the shielding side, although nulling coils are generating environments with low fields, large movements (e.g., a participant walking) have not yet been demonstrated and any movement can still generate field shifts that can obfuscate low frequency brain activity. The development of new ‘reconfigurable’ coils to place field-null volumes anywhere inside a room, and a drive to even lower remnant field, therefore remain critical areas of development. Finally, sensor ‘crosstalk’, the distortion of a field measurement at one sensor due to the presence of a second sensor, remains a challenge. For conventional single- and dual-axis sensors, this effect requires compensation, which can be accommodated in post-processing. However, in triaxial OPMs, crosstalk can be removed at source via a calibration procedure [58], again demonstrating an advantage for multidimensional measurement.

For clinical studies, there are patient groups who may be fitted with devices such as deep brain stimulators or vagal nerve stimulators (VNS). Such devices generate static magnetic fields that vary rapidly (in space) across the head. Similar fields can be generated by the more common-place presence of metalwork in the mouth (e.g., dental braces). It is extremely challenging to compensate for such high spatial frequency fields using electromagnetic coils. Conventional MEG is relatively robust to such devices, since SQUIDs have a large dynamic range. However, the extent to which OPM-MEG can cope with such situations remains unknown. That said, there are some initial positive indications. In the case of objects attached to the head, the interference fields are static relative to the sensors and so can potentially be offset by on-board-sensor electromagnetic coils [48]. For objects like VNS that likely do not move with the head, this poses a greater challenge, although potentially OPMs with closed loop operation [63] may offer a viable solution. Finally, at the time of writing, no OPM-MEG studies of participants in the age range of birth to 1 year old have been undertaken (to the authors’ knowledge). While the general promise for paediatric measurement is clear, the extent to which babies would tolerate OPM helmets in their current form is unknown. Certainly, in the first few months of life, a baby would be unable to support the weight of a helmet. It therefore seems likely that other designs would be required (e.g., a cradle, with built-in helmet, that the baby lies in). Here again, the flexibility of OPMs to be mounted and remounted in multiple devices represents a significant advantage.

These points represent some of the principal challenges faced by OPM-MEG; it is encouraging to note that although these challenges remain to been addressed, in principle, none appear insurmountable. There is reason to hope, we would argue, that despite the significant technical work that remains, the full potential of OPM-MEG to surpass conventional technology may be realised in the coming years.

## Concluding remarks

OPM-MEG systems are already gaining advantages over conventional MEG instrumentation. The advantages can be loosely classified into four areas:

- Data quality: the increased proximity of sensors to the scalp surface means that OPMs detect an MEG signal that is larger in amplitude, and better spatially localised, compared with SQUIDs.
- Adaptability: OPM-MEG can be adapted to the head size and shape of individual participants and the sensor array can be flexibly reconfigured according to the demands of specific experiments. These advantages are particularly important in paediatric imaging, where unlike conventional MEG, OPM-MEG has the potential to adapt to individuals of any age.
- Motion robustness: the ability to scan people as they move will enable data acquisition in participants who cannot tolerate the demands of current functional imaging environments, and use of new experimental designs, not possible in conventional MEG or MRI.
- System simplicity: the lack of reliance on cryogenic sensing facilitates simpler instrumentation, free from cryogenics.

OPM-MEG also has advantages over other functional neuroimaging approaches. For example, fMRI is limited to haemodynamic measurement, has poor temporal resolution, requires participants to be located in a confined and noisy environment, and requires participants to remain still, making it hard to measure brain activity in naturalistic settings. EEG and fNIRS, while enabling naturalistic activity during scanning, have either limited spatial resolution (EEG) or temporal resolution (fNIRS). For these reasons, within the landscape of functional imaging, OPM-MEG is beginning to stand out as a newly emerging tool which, in several of its characteristics, surpasses current technology.

There are a number of areas that stand to gain from these benefits. For example, increased spatial accuracy and sensitivity will be of significant utility for all functional mapping studies, including clinical applications (e.g., mapping epileptiform activity) and basic research. There is marked promise for neuroimaging in early ages: scanning babies, infants, and children is more tractable using OPM-MEG compared with SQUID MEG and this offers both clinical benefits (e.g., studying neurodevelopmental disorders) and basic research opportunities (e.g., examining how electrophysiological activity and connectivity change during the early years of life). The ability to scan during free movement opens up MEG to cohorts who would find a conventional scanner difficult to tolerate, for example, people with movement disorders. Motion tolerance also facilitates the possibility of new types of experimentation (e.g., immersive environments, or naturalistic scenarios). Finally, OPMs are not limited to the study of the brain and are finding application in measurement of electrophysiology of the peripheral nervous system, muscles, heart, and even the enteric nervous system.

In sum, this emerging technology carries the potential to provide neuroscientists with a unique noninvasive window to brain activity. Even now, OPM-MEG has significant advantages over existing techniques and, whilst technical challenges remain, there appear to be no fundamental barriers to further development and enhancement of the technology. As the field develops, this will likely generate new and exciting prospects for future, neuroimaging-based studies of brain function (see Outstanding questions).

## Highlights

Magnetoencephalography (MEG) allows noninvasive electrophysiological imaging of human brain activity. However, current MEG technology has significant limitations.

Optically pumped magnetometers (OPM)-MEG is a new type of MEG instrumentation, promising several advantages compared with conventional scanners: higher signal sensitivity, better spatial resolution, more uniform coverage, lifespan compliance, free movement of participants during scanning, and lower system complexity.

We describe the principles underlying OPM-MEG and its components, including noncryogenic field sensors and magnetic shielding technologies.

We discuss how the OPM-MEG technology is impacting neuroscience, enabling researchers to overcome limitations of conventional human imaging techniques and tackle new types of research questions.

## Outstanding questions

Can the noise floor of an OPM be reduced below that of a SQUID? This would realise the full potential for higher sensitivity across the whole brain, including deep sources.

Current wearable OPM-MEG enables free head movement but not ambulatory motion. Development of reconfigurable coil technology, where field nulls follow a subject around a room, would allow individuals to be scanned whilst they walk. This would enable new studies (e.g., examining changes in brain activity during the period when a child learns to walk). Likewise, shielding remains cumbersome and expensive. Can new types of shield be made smaller and lighter, offering easier siting in a clinical setting?

Much of the current knowledge about human brain function, particularly at the network level, comes from neuroimaging. However, the vast majority of studies involve unnatural experimental paradigms (e.g., a volunteer performing the same task many times) carried out in challenging environments. Can neuroscientists exploit the freedom offered by OPMMEG to examine brain function in more naturalistic paradigms?

Can the advantages of OPM-MEG be exploited clinically? For example, could the higher spatial resolution be used for mapping of epileptiform activity or eloquent cortex? Can the high sensitivity, coupled with the ability to develop new (naturalistic) paradigms, be used to develop better biomarkers of epilepsy or other disorders?

New technologies for neurostimulation have gained significant traction in recent years, with techniques such as transcranial electrical stimulation (TES) and transcranial magnetic stimulation (TMS) now widely available. The ability to measure (using OPM-MEG) and manipulate (using TES/TMS) brain activity simultaneously is likely to be a potent combination, begging the question, can OPM-MEG be developed to work alongside neurostimulation techniques?

## Box 1. OPM physics

At the heart of an OPM is a cell containing an atomic vapour, typically ^87^Rb (Figure I). In physics terms, the Rb atom possesses spin and hence has a magnetic moment. In the absence of external influence, the magnetic moments of the Rb atoms are aligned arbitrarily (shown by the black arrows in the upper panel of Figure I). However, if circularly polarised laser light, at a wavelength resonant with the D1 transition between quantum states (795 nm) is introduced, photon absorption causes a change in atomic energy state and an alignment of magnetic moments for all atoms in the vapour. The vapour essentially becomes magnetised, with a bulk magnetisation along the direction of the beam (Figure I, centre panel) [64]. Once in this state, the atoms can no longer absorb photons and the vapour becomes transparent to the laser light. This means the light measured at the photodiode is maximised (a ‘zero-field resonance’).

However, the bulk magnetisation of the Rb atoms in the vapour obeys the Bloch equations and so, if a magnetic field impinges on the cell (e.g., the field from the brain) the bulk magnetisation undergoes Larmor precession (Figure I, bottom panel). This reduces the net alignment of the magnetic moments and the atoms can again absorb laser light, causing a drop in light intensity at the photodetector. Light intensity consequently becomes a (Lorentzian) function of (the transverse) magnetic field in the cell [65].

In practice there are two challenges to this. First, atomic collisions cause a loss in coherence between atoms (i.e., the spin alignment is degraded). Second, a Lorentzian function is symmetric around zero field and so one cannot differentiate field direction (i.e., a positive field and a negative field of the same magnitude have the same effect). The first problem is (paradoxically) solved by heating the vapour; collisions occur so fast that coherence is not lost and spins still precess coherently, albeit at a slower rate, a scenario referred to as the spin-exchange relaxation-free (SERF) regime [6]. The second problem is solved by adding a second, oscillating, field, the ‘modulation field’, using the on-board sensor coils shown in Figure 1 in main text. The modulation field changes the solution to the Bloch equations [65] such that the photodiode output (measured using a lock-in amplifier at the modulation frequency) becomes an approximately linear function (for small fields) of the neuromagnetic field in the direction of the modulation. In other words, if the modulation field is applied in the x-direction, one can read out the x-component of the brain’s magnetic field. In practice, one can apply two modulation fields, using orthogonal signals (e.g., sine and cosine), and simultaneously measure the field along two axes (both perpendicular to the laser beam).

## Box 2. OPM helmets

OPMs are small, lightweight, and can be mounted flexibly on the head. However, the way in which sensors are housed requires ergonomic design to optimise performance and comfort. Mounting can be achieved by flexible (‘EEG-like’) caps (Figure I, left). However, flexibility can cause relative motion of the sensors during a scan, which degrades performance [54]. In addition, the location and orientation of the sensors (relative to each other and relative to the brain) is unknown and must be estimated based on some ‘coregistration’ procedure, which can be time consuming. For ‘perfect’ sensor placement and ultimate performance, it is possible to construct bespoke, solid, 3D-printed helmets (e.g., based on an MRI scan), which fit individual participants [49] (Figure I, centre). These ensure that sensors are optimally placed on the scalp for every individual. The helmet is rigid and the location and orientation of the sensors (relative to each other and the brain) is known with high precision from the (MRI-based) design and additive manufacturing process. This design is likely optimal in clinical settings; because the helmet can be removed and replaced many times, with sensors in an identical position, data can be concatenated, facilitating much longer scans. This not only increases accuracy but also increases the chances of capturing useful events like an epileptic seizure (which has been accomplished using conventional MEG [66]). However, bespoke helmets are costly, since a new one must be designed for every participant. As a compromise, generic 3D-printed helmets have been built (Figure I, right), which offer reduced cost (the same helmet can be used for many participants) and good performance (helmets come in many sizes and it is possible to take the best fitting size for each participant). Because the helmet is rigid, the relative location/orientation of sensors is known. However, a coregistration procedure is still required to determine where the helmet is relative to the brain [54].

## Box 3. Magnetic shielding

The MEG signal is around 1 billion times smaller than the Earth’s magnetic field. Interfering magnetic sources, such as those caused by passing vehicles or laboratory equipment, are also larger than the field from the brain (Figure IA). To reduce the impact of such interference, all MEG systems (including cryogenic systems) are placed in magnetically shielded enclosures (MSEs). MSEs are constructed from multiple layers of metal with a high magnetic permeability (mu-metal), which diverts lines of magnetic flux into the material, forcing them around and away from the enclosure. This shields MEG systems from both temporally static fields and low frequency interference; a separate layer of material with a high electrical conductivity screens high-frequency fields via induced eddy currents. This passive shielding provides a good environment for MEG experiments, enabling the tiny field from the brain to be measured in the absence of environmental noise.

OPM systems are advantageous since their small physical footprint means shields can be designed that are smaller and lighter than the large rooms required to house cumbersome cryogenic instruments [51]. However, inside most MSEs, a remnant static field of ~10–70 nT remains due to the presence of the metal itself. SQUIDs are insensitive to this static field, but OPMs operate around a zero-field resonance and have a low dynamic range (if the field at an OPM changes by more than ~3 nT, the device stops working). This means if an OPM rotates relative to a uniform background field, or translates in a field gradient, the cell will ‘see’ a changing background field, which would degrade its performance (Figure IB). Even if the field were low enough for the OPM to keep working (i.e., <1.5 nT), movement would mean that the OPMs measure a field shift that may obscure brain activity. For this reason, OPM-MEG systems have stringent shielding requirements, where both temporally varying fields and the remnant static field must be shielded.

These requirements have been met by combining passive shielding with active shields [33,46,47]. Novel MSEs have been constructed whereby the inner walls can be demagnetised via a process called degaussing. Using this technique, the remnant field in the room can be reduced to 5 nT, a significant improvement, but still too high for OPMs to remain within their dynamic range if, for example, a participant were to carry out a complete rotation. To tackle this, electromagnetic coils are also installed to provide ‘active’ shielding. In most systems developed to date, coils are mounted on two planes either side of the participant. A series of ‘fingerprint’ wire-paths are designed (using techniques adapted from the design of high-performance MRI scanners) and attached to the planes. Eight layers of wiring generate different types of magnetic field, including three uniform fields (along each Cartesian axis) and five first-order field gradients (fields that vary linearly with position), over a volume enclosing the head. Precision modelling [67] is used to identify the coil currents that generate a magnetic field equal in magnitude, but opposite in direction, to the remnant field (which is measured by a set of reference sensors). In this way, the remnant magnetic field is cancelled out (‘nulled’) (Figure IC). This system can also work dynamically, with constant feedback allowing drifts in field over time to be accounted for. In addition, recent work has shown that this high fidelity field control can also be exploited for calibration, as well as to identify the location and orientation of the sensors themselves [68].

At the time of writing, published work employs coils that compensate field only within a fixed volume over the head (enabling free head movement). However, emerging modular coil designs are expected to expand this capability, ultimately enabling scanning of an individual as they walk around a room.
